# The Comprehensive Management of Patients with Rhino-Orbito-Cerebral Mucormycosis; A Perspective from Antifungal Treatment to Prosthetic Rehabilitation: A Descriptive Cohort Study

**DOI:** 10.3390/tropicalmed9070158

**Published:** 2024-07-12

**Authors:** Angélica Julián Castrejón, Rosa Marene Hernández Martínez, Diana Rivero Méndez, Israel Nayensei Gil Velázquez, Juan Heriberto Rodríguez Piña, Juan Manuel Salgado Camacho, Nicolás Teyes Calva, Sayuri I. Espíndola Chavarría, Patricia A. Meza-Meneses, Carlos Alberto Castro-Fuentes

**Affiliations:** 1 Maxillofacial Surgery Service, Hospital Regional de Alta Especialidad de Ixtapaluca, IMSS-BIENESTAR, Calle Gustavo E. Campa 54, Col. Guadalupe Inn, Alcaldía Álvaro Obregón, Ciudad de México C.P. 01020, Mexico; angelica.maxilo@gmail.com; 2 Maxillofacial Prosthesis Service, Hospital Regional de Alta Especialidad de Ixtapaluca, IMSS-BIENESTAR, Calle Gustavo E. Campa 54, Col. Guadalupe Inn, Alcaldía Álvaro Obregón, Ciudad de México C.P. 01020, Mexico; pmf.marene.hernandez@gmail.com; 3 Otorhinolaryngology Service, Hospital Regional de Alta Especialidad de Ixtapaluca, IMSS-BIENESTAR, Calle Gustavo E. Campa 54, Col. Guadalupe Inn, Alcaldía Álvaro Obregón, Ciudad de México C.P. 01020, Mexico; dra.riverom@gmail.com; 4 Internal Medicine Service, Hospital Regional de Alta Especialidad de Ixtapaluca, IMSS-BIENESTAR, Calle Gustavo E. Campa 54, Col. Guadalupe Inn, Alcaldía Álvaro Obregón, Ciudad de México C.P. 01020, Mexico; hillipgv@hotmail.com (I.N.G.V.); s_yuri10@hotmail.com (S.I.E.C.); 5 Neurology Service, Hospital Regional de Alta Especialidad de Ixtapaluca, IMSS-BIENESTAR, Calle Gustavo E. Campa 54, Col. Guadalupe Inn, Alcaldía Álvaro Obregón, Ciudad de México C.P. 01020, Mexico; neurojuan@hotmail.com; 6 Neurosurgery Service, Hospital Regional de Alta Especialidad de Ixtapaluca, IMSS-BIENESTAR, Calle Gustavo E. Campa 54, Col. Guadalupe Inn, Alcaldía Álvaro Obregón, Ciudad de México C.P. 01020, Mexico; salgadocamacho71@gmail.com (J.M.S.C.); nicolasteyescalva@gmail.com (N.T.C.); 7 Infectology Service, Hospital Regional de Alta Especialidad de Ixtapaluca, IMSS-BIENESTAR, Calle Gustavo E. Campa 54, Col. Guadalupe Inn, Alcaldía Álvaro Obregón, Ciudad de México C.P. 01020, Mexico; 8 Research Unit, Hospital Regional de Alta Especialidad de Ixtapaluca, IMSS-BIENESTAR, Calle Gustavo E. Campa 54, Col. Guadalupe Inn, Alcaldía Álvaro Obregón, Ciudad de México C.P. 01020, Mexico

**Keywords:** management, rhino-orbito-cerebral mucormycosis, surgery, prosthetic rehabilitation

## Abstract

Surgical intervention is a key element in the management of patients diagnosed with mucormycosis. A retrospective cohort study was carried out, in which patients with a proven diagnosis of mucormycosis were evaluated over a period of 10 years, according to the MSGERC criteria. A descriptive analysis of the clinical characteristics, comorbidities, imaging, and microbiology studies, as well as medical and surgical treatment and the type of prosthesis was carried out. A total of 22 cases were identified, of which 54.5% (n = 12) of the population were men. Furthermore, 77.2% (n = 17) of the population had diabetes mellitus. The main antifungal treatment implemented was liposomal amphotericin B (77.2%, n = 17). The most affected structures in our patients were the paranasal sinuses (n = 18; 81%), followed by the maxilla and orbit (n = 15; 68%), nose (n = 12; 54%), central nervous system (n = 11; 50%), and skin and soft tissues (n = 2; <1%). Of the total population, 59.09% (n = 13) of patients underwent maxillofacial surgery, of which 61.53% (n = 8) required some type of prosthetic rehabilitation. Orbital exenteration and maxillectomy were the most frequent surgeries, accounting for 69.23% (n = 9), while skull base drainage was performed in four patients (30.76%). Of the total number of patients (n = 22), eight died (36.36%). Appropriate surgical management according to the affected structures, considering not only increasing the patient’s survival, but also considering the aesthetic and functional consequences, will require subsequent rehabilitation.

## 1. Introduction

In Mexico, mucormycosis is an emerging, opportunistic fungal infection that is associated with a mortality rate of more than 50%. *Rhizopus* spp. are the most frequent etiological agents in 59% of cases of rhino-cerebral mucormycosis reported in the country [1]. The most frequently identified clinical forms are the rhino-orbito-cerebral form (75.9%), the cutaneous form (8.41%), and the pulmonary mucormycosis form (7.47%), according to a 35-year retrospective study carried out in a third-level care center [2].

Among the risk factors for the development of mucormycosis, diabetes mellitus (DM) is the main one. Patients with this condition begin with symptoms such as acute sinusitis; deterioration quickly occurs with altered visual acuity, facial edema, and necrotic areas which are typical of the infection. Therefore, early diagnosis, the control of concomitant processes such as hyperglycemia, ketoacidosis, or immunosuppression, and surgery with the surgical debridement of the affected tissues are essential for the treatment of mucormycosis [3].

Imaging studies are important to determine the extent of the disease and the surgical plan to choose. The surgical management of rhino-cerebral mucormycosis is complex due to the aggressiveness of the disease and its multiple routes of spread, including unforeseen locations that have since been found to be involved [4]. Surgery is required in the prognosis of these patients, as it reduces the risk of mortality [5].

Maxillectomy is part of the approach for patients with advanced cases of the mucormycosis rhino-cerebral form; however, it alters the patient’s facial appearance, function, and quality of life [6]. Although it is known that invasive procedures as a first treatment decrease the mortality rate, the patient’s quality of life is affected [7]. Nevertheless, advances in techniques allow implants to be designed and manufactured for each patient and are of great help in rehabilitation in post-maxillectomy cases [8].

The objective of this study is to describe the multidisciplinary management of patients diagnosed with mucormycosis, including a timely diagnosis, the use of antifungals, the surgical techniques used according to the affected structures to improve the survival of these patients, as well as a prosthetic rehabilitation plan to counteract the aesthetic and functional sequelae that can decrease the quality of life of these patients.

## 2. Materials and Methods

This is a retrospective, descriptive, and cross-sectional cohort study. Patients treated between 2014 and 2024 at a single tertiary hospital center were included. The study’s population included patients with a proven diagnosis of mucormycosis according to the criteria from the European Organization for Research and Treatment of Cancer and the Mycoses Study Group Education and Research Consortium (MSGERC) [9].

The variables included were sociodemographic characteristics, clinical characteristics according to the classification of Honavar et al. [10], comorbidities, microbiological and imaging studies, medical treatment, type of surgery, type of prosthesis, and outcome. A descriptive analysis was performed for all variables. These are described as frequencies and percentages for categorical variables, and as medians and ranges for continuous variables.

## 3. Results

A total of 22 cases of patients with a diagnosis of mucormycosis were treated at the Hospital Regional de Alta Especialidad de Ixtapaluca (HRAEI). In total, 54.5% (n = 12) of the population were men. The median age is 50 years, with a range between 6 and 69 years. In total, 77.2% (n = 17) patients had diabetes mellitus, of which 41% (n = 7) presented with diabetic ketoacidosis. Only two patients (<1%) presented with any hematological disease as a comorbidity, namely leukemia (patient 5) and aplastic anemia (patient 15). The most affected structures were the paranasal sinuses (n = 18; 81%), followed by the maxilla and orbit (n = 15; 68%), nose (n = 12; 54%), central nervous system (n = 11; 50%), and the skin and soft tissues (n = 2; <1%). The diagnosis of mucormycosis was performed by means of a tomographic study compatible with mucormycosis (n = 20; 90.9%), a histopathological study (n = 18; 81.8%), and a culture (n = 1; <1%) with microscopy (n = 1; <1%). The only antifungal agent used was liposomal amphotericin B (n = 17; 77.2), implemented for an average duration of 18 days. The median hospital stay was 34.5 days, with a range between 3 and 89 days. Of the total number of patients (n = 22), eight died (36.36%) due to mucormycosis, and fourteen (63.63%) patients were discharged alive. The characteristics of the cohort are summarized in Table 1.

Based on the clinical and imaging characteristics of each patient, a multidisciplinary evaluation was performed to identify candidates for surgical and prosthetic intervention. Of those candidates, 59.09% (n = 13) underwent maxillofacial surgery, of which 61.53% (n = 8) received prosthetic rehabilitation; these patients were followed up with and had their prostheses adjusted for aesthetics and function (palatine and orbitofacial obturators). The surgical treatments performed and the types of prostheses used are described in Table 2. Exenteration and maxillectomy were the most common surgeries (n = 9; 69.23%), followed by skull base drainage which was performed in four patients (30.76%).

Depending on the severity of the disease, some patients required some type of prosthesis. Below are the cases of successful rehabilitation.

**Figure 1:**
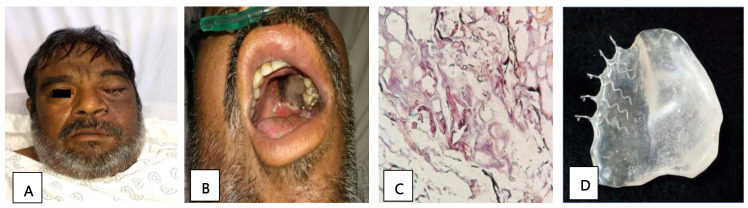
(**A**) Image showing facial asymmetry with increased volume and erythema of the left orbital and nasolabial regions. (**B**) Necrotic palatine ulcer. (**C**) Histopathological diagnosis: thick hyphae and septate at a 90° angle, compatible with mucormycosis agents (PAS stain, 40×). (**D**) Prosthetic used: transitional palatal obturator made of transparent polymethyl methacrylate with metal retainers.

Patient 2, a 51-year-old man with diabetes, presented with orbital apex syndrome (Figure 1A) with vision loss due to optic neuropathy and ophthalmoplegia due to the involvement of the ocular motor nerves, in addition to a necrotic ulcer on the palate (Figure 1B) and CNS defects. The diagnosis was made through a histopathological study (Figure 1C). He underwent a hemimaxillectomy with a skin resection of the left hemiface and exenteration of the left eye; the edges were cauterized, and an anterolateral contralateral thigh flap was performed. Subsequently, a surgical palatal obturator was placed. After seven days, the obturator was removed, and an impression was taken to create a transitional palatal obturator made of transparent polymethyl methacrylate with metal retainers (Figure 1D), and an adjustment using tissue conditioner to adequately seal the oroantral communication was carried out. Additionally, the patient received a treatment of liposomal amphotericin B and glycemic control. The edges of the surgical specimen were described as free of infection and the patient was discharged.

**Figure 2:**
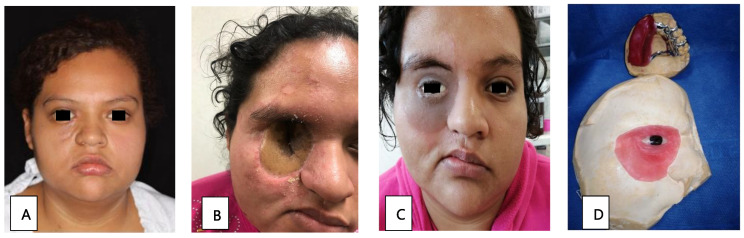
(**A**) Facial asymmetry with proptosis and orbital cellulitis of the right eye, as well as an ipsilateral malar necrotic lesion. (**B**) Radial skin graft to cover the right orbital cavity. (**C**) Placement of metal-acrylic obturator and right silicone orbitofacial prosthesis. (**D**) Metal-acrylic obturator and silicone right orbitofacial prosthesis.

Patient 3, a 22-year-old woman in the early stages of DM, presented with orbital cellulitis (Figure 2A); anisocoria, a necrotic lesion in the malar; and a 1 × 2 cm fistula on the right hard palate. A CT scan of the facial mass was performed, in which occupation on the right maxillary sinus was observed, with bone resorption on the anterior wall. Management included glycemic control, 42 days of liposomal amphotericin B treatment, and surgical intervention through a right hemimaxillectomy and orbital exenteration. During rehabilitation, the initial skin flap made had to be removed due to bacterial superinfection. A surgical ethyl vinyl acetate palatal obturator was placed, with orbital exenteration using the Ferguson-Weber approach, along with the placement of a right orbital radial skin graft (Figure 2B). An obturator adjustment was performed, and an intraoral impression was taken to change the obturator, creating a transitional polymethyl methacrylate obturator with metal retentions. Once healed, a definitive metal-acrylic obturator was made, as well as a custom silicone right orbitofacial prosthesis (Figure 2C,D). Due to the cost of treatment, it was not possible to transition the patient to oral maintenance therapy with posaconazole or isavuconazole. After the first surgery, there was no relapse of infection.

**Figure 3:**
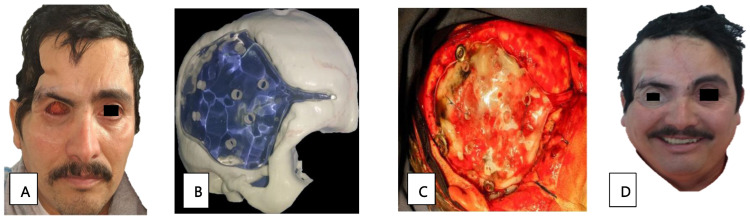
(**A**) Sequel state after exenteration of the orbit and skull base drainage. (**B**) Three-dimensional print for intracranial rehabilitation. (**C**) Cranioplasty for protein placement. (**D**) Intracranial rehabilitation and placement of a silicone orbitofacial prosthesis.

Patient 12, a 41-year-old man with diabetes, presented with paranasal, orbital, and CNS defects requiring surgical management through an orbital exenteration and skull base drainage. The prosthetic cranial and orbitofacial rehabilitation was complete. The cranial prosthetic rehabilitation was designed using a 3D-printer (Figure 3B). The impression was made of a transparent thermocurable polymethyl methacrylate for the cranioplasty, and was performed by a neurosurgeon (Figure 3C). Additionally, a silicone orbitofacial prosthesis was placed. We see a comparison of the rehabilitation in the aesthetic and functional sequelae in Figure 3A and Figure 3D, respectively.

## 4. Discussion

This study describes the comprehensive management, from antifungal treatment to prosthetic rehabilitation, carried out in patients with a proven diagnosis of rhino-orbito-cerebral mucormycosis at our hospital. Of the total cases, 77.2% (n = 17) of the population had a diagnosis of diabetes mellitus; of these 41% (n = 7) presented with diabetic ketoacidosis. In addition, hematological diseases (leukemia and aplastic anemia) were identified. The main structures affected by mucormycosis were the paranasal sinuses, followed by the orbit, nose, CNS, and the skin and soft tissues. More than half of the population required maxillofacial surgery (59.09%), of which 61.53% had some type of prosthesis placed.

The characteristics of the studied cohort reflect what is reported in the literature, in which the greatest risk factor of mucormycosis is DM [11]. However, the cohort in our 10-year study does not align with recent studies in which COVID-19 [12] hematological and post-transplant diseases are the most common risk factors [13], as we had none and two cases for hematological and pos-transplant diseases. The most common clinical presentation in our cohort was rhino-orbito-cerebral, unlike what was reported in mucormycosis associated with COVID-19 (MAC) [12], where the most common clinical presentation was rhino-orbital, found in post-transplant patients, and/or in patients with hematological diseases, whose presentation was pulmonary or disseminated [11,13]. We hypothesize that the greater number of cases of rhino-orbito-cerebral forms in our cohort has an influence on the type of surgeries performed. In cohorts where the rhino-orbital form was the most common, endoscopic surgery of the paranasal sinuses was the most frequently performed surgery [12]; this is in contrast to our cohort, where exenteration and maxillectomy were the most frequently performed surgeries. The mortality rate reported in our cohort reflects that reported in the literature, which fell between 25 and 62% in the rhino-orbito-cerebral forms, which is lower than in the pulmonary (48–87%) and disseminated forms (96%), but higher than in the cutaneous forms (25%) [9].

The diagnosis of the causal agent in our study was made mainly through a histopathological study with little recovery of cultures and direct microscopy. Tomography was the study available to determine the extent of the disease in our cohort. However, some authors recommend the implementation of magnetic resonance imaging and endoscopy as necessary additional studies to perform [14].

The management of this cohort was in accordance with the recommendations of international clinical guidelines [15]. The antifungal treatment of choice in our cohort was liposomal amphotericin B monotherapy. Because we did not have other antifungals at our center, such as posaconazole or isavuconazole, combined antifungal therapy or a transition to oral antifungals were options for our patients. According to the guidelines, surgical debridement should be carried out as many times as necessary until the antifungal agent correctly penetrates the tissue. However, to our knowledge, there are no published articles describing imaging findings that suggest that radical surgical management is insufficient to debride the infected tissue without affecting vital structures.

One of the main criteria for the surgical treatment of mucormycosis in our hospital is the progression of the disease. Most of our patients present with mucormycosis affecting between three and four structures, including the CNS; therefore, the surgical treatment is extensive. Currently, the implementation of endoscopic nasal and orbital surgeries have been reported to avoid orbital exenteration through endoscopic emptying [16]. In our study, orbital exenteration together with hemimaxillectomy was the most effective treatment to stop the disease. It is known that the debridement of all infected tissues reduces the mortality rate by 49% [17].

In our study, the Honavar classification [10] was a useful tool to determine surgical management. The most affected sites in this study were the paranasal sinuses, followed by the orbit and maxilla, nasal, and, finally, the areas that affect the central nervous system. Within the paranasal sinuses, the most affected sites were the maxillary bone, followed by the maxillary sinuses, the palate, and the palatine mucosa, which, according to Honavar et al. [10], correspond to the classifications 2c and 2d. Patients with these classifications underwent maxillectomy surgery with a turbinectomy. The second most affected site was the orbit (classifications 3c and 3d), involving the orbital vessels, damage to the optic nerve, and vision loss. These patients had to undergo orbital exenteration. The groups of patients whose presentation involved the nasal region were classified as 1c and 1d, with damage to the septum and the mucosa of the bilateral maxillary sinuses; turbinectomy was recommended. Finally, the patients with CNS defects, which correspond to classifications 4c and 4d, underwent an alteration of the cavernous sinus with the presence of brain abscesses and internal carotid ischemia, resulting from surgical treatment. However, four patients in our study underwent surgery to drain abscesses, because they did not yet have neurological deficits and because the patients requested surgical treatment. Of these, one patient was discharged, and died a few days after the intervention.

In our cohort, some patients were not eligible for surgical management because the severity and extent of the disease did not allow for it; such was the case for patient 19, whose magnetic resonance image confirmed sinus thrombosis in the ipsilateral cavernous artery and involved the optic nerve, as well as an ischemia in the middle cerebral artery associated with thrombosis in the right internal carotid artery. Therefore, she was discharged for her maximum benefit and did not receive surgical treatment.

Due to the extent of the large hemifacial defect, for patient 2, a flap of skin and femoral muscle had to be removed and anastomosed to the facial vessels, as reconstruction by means of a prosthesis was limited. As reported by Alfano et al. [18], who performed a pectoralis major pedicled myocutaneous flap surgery for the reconstruction of an exenteration, the excellent blood perfusion in the patient allowed for antifungal action. It has also been suggested that free flaps may play a role in delaying reconstruction [18]. Murphy et al. [19] reconstructed an extensive hemifacial defect with an anterolateral free thigh flap, incorporating a segment of the vastus lateralis to erase the dead space left by the debridement of the paranasal sinuses.

It is important to consider that the objectives of reconstruction may vary in each case, such as providing coverage for open wounds, the obliteration of dead spaces, the closure of fistulas, and the restoration of skeletal structures; all aim to restore nasal breathing, oral nutrition, and speech. Reconstruction in 61.53% of the surgical cases in this study required the management of a trans-surgical prosthetic, such as a surgical palatal obturator or the partial skin coverage of a cavity, as reported in other cases [20]. Although most of the literature agrees on the wait-time for a reconstruction (one and a half months), in our case, most of our patients already had sufficient antifungal treatment and could not leave the cavity open as to avoid a post-surgical infection. Therefore, it was discovered that it was possible to perform flap rotation surgeries and emergency surgical obturators, and, as a result, reconstruct the maxilla or orbit based on what is recommended in the literature [20,21,22].

There are a few studies that report the consequences faced by patients with rhino-orbito-cerebral mucormycosis and how it affects their quality of life. The main sequelae are facial disfigurement (18.4%), a chewing/swallowing disability (17.8%), a language/articulation disability (14.7%), blindness (9.8%), and decreased visual acuity (10.8%) [12]. Chouksey et al. [23] studied the quality of life of 32 patients undergoing maxillectomy for mucormycosis, applying the OHIP-14 (Oral Health Impact Profile) questionnaire, finding very high pre-rehabilitation scores that substantially improved post-rehabilitation (52 vs. 6.5). Due to the retrospective nature of our cohort, we did not have the opportunity to objectively evaluate how the surgical sequelae affected our patients; however, due to the high rate of exenterations and maxillectomies performed, we can infer that these were important and affected the quality of life in our patients.

**Figure 4:**
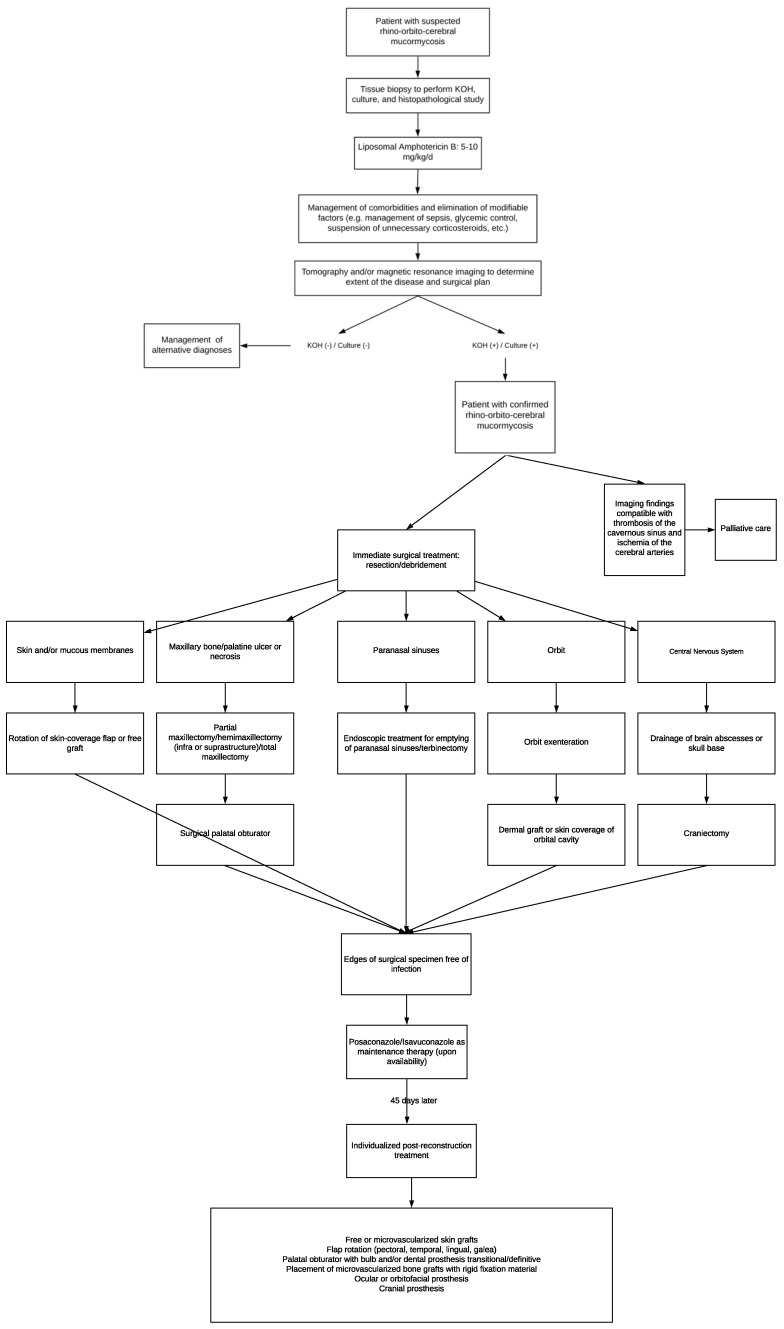
Algorithm about initial approach, medical, and surgical treatment for mucormycosis patients.

Due to the above and considering that the management of patients with mucormycosis must be conducted according to the availability of surgical techniques and regional antifungal treatments, we propose the following: an algorithm where the initial approach, medical, and surgical treatment, as well as rehabilitation, are integrated to increase the survival rate and improve the quality of life in these patients (Figure 4).

The main limitation of our study includes its small number of patients. Our hospital is a specialty center that treats serious and complex cases; therefore, it is not surprising that half of our cohort already had CNS defects at the time of diagnosis, which made creating a surgical plan difficult. Therefore, recognition of this disease at the first and second levels of care is essential for the prompt referral of the patient and timely surgical intervention.

Mucormycosis is a disease associated with a high mortality rate and serious sequelae, so it is necessary to design more longitudinal studies to determine the appropriate surgical management according to the affected structures, and that consider not only increasing the patient’s chance of survival, but also the aesthetic and functional consequences of surgery that will require rehabilitation later. Patients with rhinocerebral mucormycosis must receive multidisciplinary evaluation as by integrating the data from all clinical studies, the stage of severity of the infection can be determined and thus the type of surgical debridement most indicated for each case can be planned, followed by rehabilitation. For large defects, there are multiple forms of reconstruction that can be applied in each case if there is a safety margin to determine that the patient is free of infection, since it is necessary to continue with antifungal treatment after reconstruction.

## 5. Conclusions

Of the total population in this study diagnosed with mucormycosis, 54.5% were men. Diabetes mellitus was the main comorbidity identified in our patients, accounting for 77.2%. The main antifungal treatment implemented was liposomal amphotericin B (77.2%). The most affected structures in our patients were the paranasal sinuses (n = 18; 81%). In total, 59.09% (n = 13) of patients underwent maxillofacial surgery, of which 61.53% (n = 8) required some type of prosthetic rehabilitation. Orbital exenteration and maxillectomy were the most common surgeries performed, at 69.23% (n = 9), followed by skull base drainage in four patients (30.76%), and death in eight (36.36%).

Maxillofacial surgery is essential in severe cases of mucormycosis, particularly in the rhino-orbito-cerebral clinical form. Additionally, the placement of maxillofacial prostheses improves the quality of life in patients with craniofacial defects due to mucormycosis.

## Figures and Tables

**Table 1:** Clinical characteristics of the study’s cohort with a diagnosis of mucormycosis.

PATIENT	SEX	AGE (Years)	DM	CLINICAL PRESENTATION OF MUCORMYCOSIS					DIAGNOSIS (HISTO/CT/KOH)	ANTIFUNGAL	HOSPITAL STAY (Days)	OUTCOME
				NASAL	PARANASAL	ORBIT	CNS	OTHER ORGANS AFFECTED				
1	F	41	YES	0	2c	3c	4d	NO	HISTO/CT	LAmB	39	DECEASED
2	M	51	YES	0	2c	3d	4d	NO	HISTO/CT	LAmB	31	ALIVE
3	F	22	YES	0	2c	3c	0	NO	HISTO/CT	LAmB	58	ALIVE
4	M	59	YES	1d	2d	3c	0	NO	HISTO/CT	LAmB	89	ALIVE
5	M	69	YES	0	2c	0	0	NO	HISTO/CT	LAmB	45	ALIVE
6	M	51	YES	0	2d	3c	0	NO	HISTO/CT	LAmB	8	ALIVE
7	M	45	YES	1d	2d	3d	4d	NO	HISTO/CT	LAmB	33	DECEASED
8	F	50	YES	1d	2d	3c	0	NO	HISTO/CT	LAmB	6	DECEASED
9	F	64	YES	1d	2d	3c	4d	NO	HISTO/CT	None	0	ALIVE
10 *	M	68	YES	ND	ND	ND	ND	ND	HISTO	None	0	ALIVE
11	F	25	NO	1c	2c	3c	4d	NO	HISTO/CT	LAmB	90	ALIVE
12	M	41	YES	0	2c	3c	4c	NO	HISTO/CT	LAmB	79	ALIVE
13	M	6	NO	0	0	0	0	YES	HISTO/CT	None	3	DECEASED
14	F	45	YES	1d	2d	3c	4d	NO	HISTO/CT	LAmB	49	ALIVE
15	F	47	YES	1d	2d	3d	4d	NO	HISTO/CT	LAmB	43	DECEASED
16	M	35	YES	1	2d	3c	4d	NO	HISTO/CT	LAmB	14	DECEASED
17	F	57	YES	1c	2d	0	0	NO	HISTO/CT	None	13	DECEASED
18	M	58	NO	0	0	0	0	YES	HISTO/CT	LAmB	5	DECEASED
19	F	34	YES	1	2c	3c	4c	NO	HISTO/CT	LAmB	12	ALIVE
20	M	50	YES	1c	2d	3d	4d	NO	CT/KOH	LAmB	5	ALIVE
21	F	57	ND	ND	ND	ND	ND	ND	ND	ND	ND	ALIVE
22	M	53	NO	1d	2c	0	0	NO	CUL/CT	LAmB	36	ALIVE

F—female; M—male; DM—diabetes mellitus; CNS—central nervous system; HISTO—histopathology; CT—computerized axial tomography; KOH—potassium hydroxide; LAmB—Liposomal Amphotericin B; ND—not determined; 1c—nasal septum; 1d—bilateral nasal mucosa; 2c—more than two ipsilateral sinuses and/or oral cavity or palate; 2d—bilateral sinuses or zygoma or mandible; 3c—occlusion of the central retinal artery or ophthalmic artery or thrombosis of the superior ophthalmic vein or involvement of the superior or inferior orbital fissure orbital apex or vision loss; 3d—bilateral orbital involvement; 4c—involvement beyond the cavernous sella, involvement of the base of the skull, occlusion of the internal carotid artery or cerebral infarction; 4d—multifocal or diffuse disease of the central nervous system; *—The patient refused to be hospitalized for management of mucormycosis.

**Table 2:** Surgical management and prosthetic rehabilitation used in patients undergoing surgery.

PATIENT	SEX	AGE (Years)	SURGERY	SURGICAL TREATMENT					PROSTHESIS	TYPES OF PROSTHESES
				MAXILECTOMY	FLAP	TURBINECTOMY	EXENTERATION	SKULL BASE DRAINAGE		
1	F	41	YES	YES	YES	YES	YES	NO	NO	
2	M	51	YES	YES	YES	YES	YES	NO	YES	PALATAL OBTURATOR (SURGICAL AND TRANSCISIONAL)
3	F	22	YES	YES	YES	YES	YES	YES	YES	PALATAL OBTURATOR (SURGICAL, TRANSCISIONAL, AND DEFINITIVE), ORBITOFACIAL PROSTHESIS (EYE AND EYELID)
4	M	59	YES	NO	NO	NO	YES	NO	YES	EYE IMPLANT
6	M	51	YES	YES	NO	YES	YES	NO	NO	DOES NOT APPLY
11	F	25	YES	YES	NO	NO	YES	YES	YES	
12	M	41	YES	NO	YES	NO	YES	YES	YES	CRANIAL AND ORBITOFACIAL PROSTHESIS (EYE AND EYELID)
13	M	6	YES	NO	NO	NO	NO	NO	NO	
14	F	45	YES	YES	NO	YES	YES	YES	YES	PALATAL OBTURATOR (SURGICAL AND TRANSCISIONAL)
15	F	47	YES	NO	YES	NO	NO	NO	NO	
18	M	58	YES	YES	YES	YES	NO	NO	NO	
21 *	F	57	YES	YES	NO	NO	YES	NO	YES	TRANSCISIONAL PALATAL OBTURATOR AND ORBITOFACIAL PROSTHESIS (EYE AND EYELID)
22	M	53	YES	YES	YES	NO	NO	NO	YES	PALATAL OBTURATOR (SURGICAL AND TRANSCISIONAL)

F—female; M—male; *—Patients who underwent surgery at another hospital and received prosthetic rehabilitation at our hospital.

## Data Availability

Data are contained within the article.
